# 

**Published:** 1887-07

**Authors:** 


### CONTENTS, JULY, 1887—VOL. 3, NO. 1.

ORIGINAL COMMUNICATIONS.—Imperforate Hymen, a Case; C. O. Weller, M. D., Austin.......................... 1
Notes from Obstetric Practice, Dr. Ado. Betz, Germany.. 3
Phenomenal Case of Hysteria, F. A. Schmitt, M. D., Shulenburg........................................ 6
Cocaine in Cautery of Venereal Warts, D. Berry, M. D., San Antonio........................................ 10
A case of Quadruplets, S. T. Lowry, M. D., San Antonio. 12
A Note on Electrical Treatment, F. T. Paine, M. D., Comanche........................................ 12
CORRESPONDENCE.—A Spicy Letter to the B. M. and S. Journal........................................ 13
Urethan in Fever—Note from Dr. Beall.................. 15
SOCIETY NOTES.—East Texas Medical Association........ 16
Travis County Medical Association..................... 16
EDITORIAL.—The Texas Medical College and Redivivus.... 17
A Rape on Science.................................. 18
Texas Delegate and the International Congress......... 20
MEDICAL NEWS AND MISCELLANY.—Twenty-eight Items of Interest........................................22 to 27
ADVERTISERS' NOTICES.—Numerous........................28 to 30

BOOK NOTICES, of which we had a large lot ready, are omitted for want of space. Beginning a new volume with a large accession of advertisements, we felt we must notice them all.—ED.

